# The Weakening of Kin Ties: Exploring the Need for Life-World Led Interventions

**DOI:** 10.3390/ijerph15020203

**Published:** 2018-01-25

**Authors:** Gert Schout, Gideon de Jong

**Affiliations:** 1 Department of Medical Humanities, VU University Medical Centre, P.O. Box 7057, 1007 MB Amsterdam, The Netherlands; 2 Edith Cowan University, School of Nursing and Midwifery, Building 21, Room 21.423, 270 Joondalup Drive, Joondalup, WA 6027, Australia; g.dejong@ecu.edu.au

**Keywords:** Family Group Conferencing, life-world led interventions, thick and thin solidarity, social embeddedness, kin ties, Sloterdijk’s sphere theory

## Abstract

The protective features that families and wider social relationships can have are required to meet the demands of life in contemporary Western societies. Choice and detraditionalization, however; impede this source of solidarity. Family Group Conferencing (FGC) and other life-world led interventions have the potential to strengthen primary groups. This paper explores the need for such a social intervention, using insights from sociological and philosophical theories and empirical findings from a case study of the research project ‘FGC in mental health’. This need is understandable considering the weakening of kin ties, the poor qualities of state agencies to mobilise self-care and informal care, its capacity to produce a shift of power from public to private spheres and its capacity to mitigate the co-isolation of individuals, families and communities. A life-world led intervention like FGC with a specific and modest ambition contributes to small-scale solidarity. This ambition is not inclined to establish a broad social cohesion within society but to restore; in terms of the German philosopher Peter Sloterdijk; immunity (protection) and solidarity in primary groups, and consequently, resolve issues with those (family, neighbours, colleagues) who share a sphere (a situation, a process, a fate).

## 1. Introduction

The importance of families in adapting to new norms, addressing cultural traditions, providing socialisation, and helping to cope with daily challenges is beyond doubt; however, several scholars have suggested, in recent years, that family life can also be a source of stress [1], a battle ground for competitive co-parenting [2] or a trial in balancing parental goals and adolescents’ desire for autonomy [3]. Gazso and McDaniel [4], on the other hand, see families as a necessity for dealing with the constraints and structures of the neoliberal welfare state. How essential, and at the same time, difficult, family life may be, kin ties in contemporary Western societies are weakening [5,6,7]. In a paper on evolutionary sociology, Maryanski (in [8]) challenged “some long held beliefs in sociology about humans’ needs for extended kinship, intimate ties, collectivism and solidarity, arguing that human nature is, in fact, predisposed towards freedom, autonomy, restricted kinship networks, weak ties, and mobility in space” [5] (p. 419). Thick solidarity of close kinship bonds and collectivism is connected to existential hardship [9,10]. Conversely, thin solidarity is linked to individualism and the absent of such hardship [11]. The weakening of kin ties has created space for ambivalent commitments: the choice to support each other is feasible as much as the possibility not to do so. The social structures of solidarity are easily undermined, in spite of shared life events, but these structures can also be restored. Shared life events are not yet always possible because of geographic distances between family members and the changes in family composition, due to the transformations of family arrangements associated with divorces and migration [12,13].

Where Bauman [14,15] describes how the loss of solidarity that comes with the context of individualisation and detraditionalisation is connected to the ‘liquidity’ of love and life, Gazso and McDaniel [4] underline the protective features that families and wider social relationships have in dealing with the uncertainties of life in late modern societies, especially under neoliberal conditions. This raises the need for the strengthening of families and near communities in dealing with the precarity these conditions bring. Since 2009, an ongoing study has been conducted in The Netherlands in an attempt to reach out and reunite extremely marginalised people with their primary groups, or what is left of them, using Family Group Conferencing (FGC). FGC brings the social networks of family members and professional stakeholders together in a family-led decision-making forum that enables every participant to be heard and provides prospects for solutions. Since its implementation in public health systems of various Western countries in the 1990s, many researchers have attempted to examine FGC’s effectiveness (see for an overview [16]), although its theoretical foundations have remained under-researched. The aim of this paper is to understand the need for FGC in contemporary Western societies as a life-world led intervention that combines voice, self-organisation and professional support. The principles of such a life-world led practice comprise understanding the community members’ life-world views and their views of their potential, offering resources and facilitating empowerment, sharing life-world case studies, and lobbying to influence local and national policy, in relation to both the individual and communities [17].

In the following section, sociological theories that shed light on the need for the strengthening of primary groups and the prospects that a life-world led intervention such as FGC might offer will be discussed. That such interventions hold potential to generate solidarity on a small-scale is illustrated by a case study from the research project ‘FGC in mental health’. The case study reveals that sociological theories alone are not sufficient to grasp the need for FGC in contemporary Western societies. In the discussion, therefore, the ‘sphere theory’ of the philosopher Sloterdijk is brought in for its explanatory value of why life-world led interventions hold promise for regaining small-scale solidarity.

## 2. Background

Influential scholars, like Fukuyama [18,19], Pinker [20], Siedentop [21] and Shermer [22], describe a broad development across the Western world towards social justice, democracy, horizontal relationships and individualism. Once a context of social justice and democracy is established, the role of kin ties becomes less important to keep the wicked world outside. Shermer [22] points out how kin altruism has evolved in reciprocal altruism and the blind altruism of modern society. He signals the rise of reasoning, deliberation, horizontal relationships, and a much safer and less violent world. Despite the precarity of life under neoliberal conditions for some, the family as a safe haven in a dangerous world has faded away into the background. This, however, has led to a weakening of kin ties [5,6,7].

### 2.1. The Weakening of Kin Ties

Following Pahl and Spencer [23], Gazso and McDaniel [4] use the concept of “personal communities” to describe that family ties are not only given, but also chosen, and that ties can be adjusted, created and maintained by individuals. They postulate personal communities mainly as “communities of choice” and conclude that families nowadays are increasingly families by choice [4]. With sound arguments, Gazso and McDaniel [4] conclude that family ties in contemporary Western societies are no longer immediately determined nor conditioned by blood, tradition or history and that choice concerning the degree of intimacy and mutual support can be made individually. Processes of individualisation, detraditionalisation and secularisation deter individuals from collective enterprises. Lorentzen and Hustinx [24] describe this process as a shift from ‘communities of fate’ to ‘communities of choice’, meaning that collective identities are replaced by subjective individualism—processes where individuals constitute their own life more or less independent of the collective.

Where Gazso and McDaniel [4] highlight the protective features that families and wider social relationships can have in dealing with the uncertainties of life, Bauman [14,15] articulates the loss of solidarity that comes within the context of individualisation and detraditionalisation, in terms like ‘liquid love’ [14] and ‘liquid life’ [15]. The background of liquid modernity [25] does not easily generate durable mutual support or loyalty within families, although the precarity of insecure employment and unbelonging under neoliberal conditions indeed requires such bonds.

Social groups can be distinguished as given and chosen. In contrast to given communities, you can choose to leave a chosen community. Hirst [26] (p. 52) sees a community of fate as an ‘existential community’: one is born and raised in it, actors share a situation, a process or a fate. Boundaries, identity and belonging are crucial features of both communities. At first sight, these features of communities of fate are the point of application for a life-world led intervention such as FGC. Nonetheless, its connotations are problematic. Where communities of choice postulate the liberated individual—a person free from obligations and able to choose from all options that markets have to offer—communities of fate articulate bonding and the constraints of fate. Acts of solidarity are, contrarily, expressed in situations that are given and chosen at the same time. No one has chosen their family, nor the place of their cradle. Still there is freedom to maneuver and to shape their life project within different ratios of given and chosen relationships. For good reasons, sociologists designate institutions like families and friendships as natural, tacit and powerful sources of regulation. Pahl and Spencer [23] view peoples’ lives as embedded in active and significant network ties that are both given and chosen. The chosen relationships, according to Pahl and Spencer, include kin and non-kin. They refer to these social structures as ‘personal communities’. This seems, nevertheless, a contradiction in terms. A community is, and never can be, personal; it is the ultimate shared life-world, where others pull the strings as well. For this reason, in this paper, concepts like ‘families of choice’, ‘personal communities’ or ‘communities of fate’ are avoided; instead, the term ‘primary groups’ is used to underline the exchanges of implicit items, such as love, caring, concern, animosity, support—incorporating the protection of given, chosen and acquired relationships they can offer.

It is necessary to add a caveat here. Moreno-Mínguez, Martínez-Fernández and Carrasco-Campos [27] point out that in Southern European countries, where family policies have been residual, the family is still the main agent for providing inter-generational care. Naldini and Jurado [28] seem to agree that Southern European countries were, until recently, ‘familialistic’, but that also these countries have moved from the family/kinship model towards a dual-earner family model, constituted by national policies. Although there are exceptions of countries where the family/kinship solidarity model remains powerful, such as in Italy, the reasoning in this article refers to Western societies.

### 2.2. The Great Disembedding

The discussed theories shed some light on the rise of individualism and the changing role of kin ties, but are still not sufficient to convey the need for strengthening families. For this, the insights of Charles Taylor are called in. Taylor [29,30] refers to the great disembedding as the loosening of ties of clans, communities and the mandatory customs, traditions, rituals and religious obligations that come with it. Before modernisation, people could not imagine themselves outside their social context. ‘How would it be if I would emigrate?’ or ‘What would happen if I convert to another religion?’, that one can ask such questions nowadays refers, according to Taylor [29] (p. 60), to this disembedding. The great disembedding, with its “unprecedented primacy to the individual” [30] (p. 146), marks the end of the “triple embeddedness, where human agents are embedded in society, society in the cosmos, the cosmos in the divine” [30] (p. 152). Following Tocqueville’s description of ‘Société des ordres’, Taylor [29] describes a division between societies, where hierarchy and mediated access are dominant and societies in which horizontal relationships with direct access prevail. Before modernisation, one always belonged to a part of a hierarchically-ordered chain, a chain in which a farmer was connected to a feudal lord, who, in turn, was indebted to the king. Families were actually a kind of large household in which also people who were no relatives lived together with the core group: employees, students, a cousin who came to learn a trade, etc. [29] (p. 148). These households were strongly patriarchal under the undisputed authority of the male head. Dependency relationships and hierarchy were paramount. Everyone knew their place; hierarchical and mediated relationships determined the image [29] (p. 149). In modern society, traditional vertical ordinances to establish safety are replaced by modern horizontal ordinances expressed in ideas of self-consciousness, self-determination, self-realisation and self-assertion [31]. In modern horizontal societies, everyone has direct access and equal distance to the center of power, marked by an impersonal egalitarian organisation, where citizens have a direct relationship with the state. But, as Habermas [32] demonstrates, in contemporary Western societies the state has become increasingly disconnected from its citizens.

### 2.3. Market-Forces in Health Care and the Disconnecting of Life-World and System-World

The rise of commerce is linked to the rise of social justice. The work of Fukuyama [18,19,33], Shermer [22,34] and Pinker [20] demonstrates how commerce, competition, individualism, democracy and social justice recall each other and ultimately lead to less violence and more security. The rise of commerce in health care, however, has side effects that are related to commodification. Pellegrino [35] underlines that the commodification of health care means that the relationship between clients and professionals becomes a commercial relationship, which legitimises the rules of commerce and the pursuit of self-interest. In a market-driven (neoliberal) health care system, commodities are fungible; professionals can be substituted for any other similar commodity, meaning that provided quality and price are the same. In this view of health care, physicians and patients become commodities too. Until the 1980s, social workers, general practitioners and community (mental) health nurses in most Western countries knew families and their social surroundings well, accompanying their illnesses, life events and worries. Professionals stayed close, knowing that they depend on trust that stems from intimate personal relationships. Nowadays, in the market-driven health care system, health care providers look for ways to expand their services. The incentives of the market hinder the activation of self-care and informal care [36]. The professional dominance undermines people’s self-confidence and their capacity to solve problems themselves [37].

The profusion of rules and protocols for the purpose of replicable treatment, accountability and efficiency to establish an equal playing field for market players, widens the gap between what Habermas [32] calls the life-world and the system-world. The central problem of contemporary societies, according to Habermas, is how to create conditions for what he refers to as ‘communicative action’, which is the process of reaching a common understanding. Core of his Theory of Communicative Action is the ‘colonisation of life-world by systems’, meaning that the private sphere is penetrated by the strategic communication of systems in the public sphere. The system-world is characterised by rationalisation, efficiency, calculability, control and predictability. Central to the life-world is a common understanding and a shared sense of identity. It is the latent sum of all sorts of assumptions about our identity, values, desires and beliefs. In the life-world, values, achievements and beliefs are constantly reaffirmed. Habermas observes colonisation processes throughout society. In Habermas’ view, communities have fewer spheres for communicative action than state agencies. In other words, the community of generalised others is transforming into a contract-based state of taxpayers without responsibility for each other. Communicative action deals with creating a so called ideal ‘speech situation’: a situation free from coercion, where every subject is allowed to speak, to question, to express and to assert; a situation wherein the colonisation of the life-world by systems is pushed back. The logic of the system tends to overpower people’s logic. Burns and Früchtel [38] underline that FGC is needed to cure the unhelpful side effects of a legal and professional welfare state system.

### 2.4. Family Group Conferencing

Drawing on Habermas’ critical social theory, Hayes and Houston [39] re-work aspects of FGC processes, by emphasising the possibility of an empowering dialogue between families, who embody the life-world and professionals, who represent the system-world. They project Habermas’ theory as a way to constitute a moral practice by communicational procedures that address issues relating to the use of power and the need for recognition between subjects.

Although there are different approaches and applications of FGC in different contexts, there is consensus on its core features [40]. FGC is a facilitated group dialogue where citizens take matters into their own hands, solve problems and make plans following a structured decision-making process. Professionals contribute but their role in FGC is not to make decisions. Professionals rather facilitate decision-making, by providing information, resources and expertise [39]. FGC resembles the form that problem-solving takes in traditional societies, in which families or small communities handle their own issues, rather than exporting them into the hands of professionals [41].

The core idea of FGC is a meeting of all family members, state officials, and other persons who are involved with the family, in order to establish a plan for the care and protection of individual family members. The meeting is organised by an independent coordinator, who creates conditions so that the primary group can come to terms and find solutions during a private time where professionals are not present. Everyone who can contribute is invited to participate—not only family members, but also friends, neighbours and community members, such as teachers and sports coaches. Through a democratic process the family establishes its own plan on which everyone needs to agree.

Since its introduction in the 1980s in New Zealand, FGC is increasingly organised in child protection cases across the Western world [41]. More recently, in various countries, a broader perspective of situations where FGC could be implemented has taken over, which emphasises not only the capabilities of families, but of the civil society in general. The decision-making process of FGC is applied in all sorts of situations where social groups take matters in their own hands. Examples hereof are the organisation of FGC for social assistance recipients [42] and in elderly care [43]. In a study of FGC in Dutch mental health care, promising applications of FGC, with different target groups, are seen, such as with multi offenders, homeless youth, residents at risk of eviction and psychiatric patients who are threatened with coercion. But, it is also used in situations of nuisance and liveability problems in neighbourhoods, where so called community conferences are used [44,45]. Extremely marginalised people with multiple health and social problems seem to benefit from the reunion with their relatives.

The underlying philosophy of FGC assumes that primary groups are able to find workable solutions, whether or not supported by the expertise of professionals. In contrast with traditional intervention models, FGC encourages the family to determine the agenda. FGC is sensitive to the culture, lifestyle and history of families. It achieves results through the family and therefore uses the resources that exist within communities, although the role of professionals (such as social workers, community [mental] health nurses and general practitioners) does not cease to apply. Professionals refer clients to FGC, they are present during parts of the conference and they provide information and support, but also open up resources from other agencies. In cases of severe and ongoing health and social problems, research has shown that families and near communities are willing to get involved, but only when the continuity of care of professionals is guaranteed [36,45]. This sheds light on a new perspective on social work and community mental health practices, namely work that is done by various actors from the civil and professional society.

## 3. A Case Study of FGC

From 2011 until 2015, the process and impact of 82 family group conferences in different mental health care settings in The Netherlands [44,45] were examined. Findings indicate FGC’s potential in the recovery of kin ties, the regaining of ownership and the restoring of belongingness.

The 82 conferences were retrospectively reconstructed using Stake’s [46] (multiple) case study approach. Responsive methods [47,48] were used to provide a platform for clients, their social networks, professionals and FGC coordinators, to describe process and impacts of the conference from their perception.

The case study highlighted in this paper is derived from this project. The interviewed family consisted of two adult sons (both in their twenties), their mother and stepfather. Besides these four interviewees, a stepsister (daughter of the stepfather), a social worker (a man in his fifties who was employed with the local social welfare services) who became involved with the family in early 2012 and who referred the family to FGC, and the FGC coordinator, were interviewed. In total, seven interviews were held, that lasted, on average, 60–90 min. The interviews were audio-recorded, additionally transcribed and analysed, with the help of ATLAS.ti. A group member check was organised to validate the interview findings [49].

The reason for highlighting this particular case is that it demonstrates how families in contemporary Western societies can lose each other, and, as they are not socially embedded in near communities, they lack social support that extended family members, friends and neighbours might have to offer in the upbringing of their children. The case does not stand for contemporary Western family life, but it holds exemplary value, in the sense that social work and community mental health practices with similar challenges (families facing multiple problems who lack social support) can extract lessons from this case study when organising FGC [50].

## 4. The Case—Two Troublesome Adult Sons and Their Uncertain Parents

A family with two adult sons (27 and 25 years) is living in a regional area in the north of The Netherlands with an overrepresentation of persons with a low socioeconomic status. Both sons are still living at home. They have been unemployed for a while, use drugs and alcohol excessively, and cause a lot of trouble (petty crimes and oppressive behaviour). Above all, they are terrorising the lives of their mother and stepfather. The situation gets out of hand when, during a barbeque party in the back garden, the garage is set on fire and the mother’s jewels get stolen. A social worker is appointed to help in solving the problems. He quickly realises that individual care trajectories for the family members probably would not help in reaching a settlement. Besides, the mother is anxious and afraid that she will be stigmatised when referred to mental health care. According to her, the problems are interrelated. It is mainly both sons who contribute to the problematic situation, so she questions if there is any sense for individual trajectories:

That needs to be solved here! You do not want to burden other people, but problems need to be shared with your family as they are more closely related. […] They knew that the situation got out of hand.

(mother)

As the situation is so threatening, the social worker wants a family group conference to be organised in the short-term. The conference can be quickly deployed as there are no waiting lists for this type of service. The conference makes it possible to discuss the problematic situation from different angles as, besides of the sons and their parents, their significant others are also invited for the conference. An aunt (sister of the mother) and uncle and two stepsisters (children of the stepfather) agree to participate.

Prior to the conference, the parents tried numerous times to solve the problems during informal discussions with their sons at the kitchen table when having dinner. These discussions, however, quickly turned into conflicts, and, in addition, the sons left the dinner angrily. As the family group conference is organised in a neutral environment, namely a community centre, in the presence of a wider circle of bystanders (the extended family), it is more difficult for the sons to leave the discussion, in comparison with participating in an informal situation with just their parents. Both sons act indifferently towards the conference, but finally decide to participate, as they feel pity for their mother and, to a lesser degree, their stepfather. The purpose of the conference is to establish a plan to help the sons to find accommodation on their own and a paid job, so that the mother and stepfather are relieved from troubles and worries.

The conference is organised four weeks after the referral. The private time of the conference lasts for a long time and is very intense. At a certain moment, it becomes very emotional for the mother, so she bursts into tears. Subsequently, a sister of the mother and the daughter of the stepfather confront both sons with the misery they are causing to their mother and stepfather:

I said to them: “When are you both finally gonna say sorry to my sister? When are you finally gonna do so? You have treated her like an animal! Why are you making the life of my sister such a misery? Where do you think this will contribute to?”.

(sister of the mother)

My stepdaughter also started yelling: “Why are you not working? Why is the police all the time coming over? Why are you troubling the life of my father and your mother?” That was really tough, but very necessary.

(mother)

This is the moment when the sons finally realise the impact of their behaviour:

When my mother started crying, I felt pity for her. And finally, I thought: something needs to be changed, because I have never heard my mother’s side of the story. At home, you always leave the kitchen table angrily and you never hear of the other side. […] I looked at my brother, and thought ‘shit’, we need to convince it all to them. Then, they got to know why we had so many debts. Well, that was because of cannabis use, buying liquor, partying every night. […] Before I always kept this secret, I never told anyone about it. […] And when I finally told them, it felt like a relief.

(youngest son)

It appears that because of the presence of extended family members, the sons do not dare to leave the meeting. Their aunt and uncle and stepsisters function as ‘shock absorbers’, preventing the conference from stalling. As they confront the sons with their behaviour (different viewpoints that were not shared during the informal meetings at the family’s kitchen table), the sons finally start to realise that something should be changed for the better.

One fundamental principle of FGC is that participants should not only be the owner of their problems, but also become the owner of the potential solution, by establishing their own plan. Ownership of the process is feasible when professionals dare to let the primary group make up a plan to reduce risks. Nevertheless, this is not an easy task in threatening situations. Professionals provide information and support the participants in reaching a plan. When a plan is established, professionals can review whether or not it is realistic and plausible that the threatening situation will no longer occur.

During the preparation and the conference itself, the social worker radiates trust in the family’s capacity to come up with a workable plan. At the end of the conference, he assesses whether all problems are covered and concludes that the conference has more potential to reach an overall solution than individual care trajectories. In the case of this family, individual trajectories, according to the social worker, could never had the same impact and would have cost a fortune:

In the end, one should make a calculation. Think about the situation when this method was not deployed, what would have been the risks afterwards? The mother would have been send to the local mental health clinic, and the police would have been involved sixteen times more! That are just Euro marks!

(social worker)

The impact and the quality of the plan is linked to the role and the position of the coordinator, a man who is originally from the same region as the family and who works in his professional life as a piano tuner. His role as an independent fellow citizen is highly appreciated by the family and the social worker. During the preparation, the coordinator is accessible, even during the night time and in weekends, and he has a non-judgmental attitude towards the situation. In this case, as well as in other cases from our research project, it seems that clients who have a troubled history with representatives of the professional society, have less trouble with a fellow citizen. As coordinators are independent and free of ties with agencies, they can also use words that could never be used by professionals. Illustrative is a memo made just before the member check meeting with the participants of the case that was organised after analysis of the individual interviews. This member check should have initially been held in a community center in order to validate intermediate findings from the interviews:

At the supreme moment, the family does not show up [we—researchers and FGC coordinator—are waiting for them in a local community center not far from their home]. When we call them and try to convince them to come over, they act reluctant. The coordinator immediately takes over the telephone and tells the mother in her own dialect [Gronings] that it is very rude to let people come over from far and not showing up themselves. We get into the car and ten minutes later we evaluate in informal circumstances at their kitchen table the positive outcomes of the conference.

(empirical memo)

Shortly after the conference, the oldest son starts living with his girlfriend and finds a job. The youngest son is still living with his mother and stepfather, but is no longer causing trouble and has found a job as well. Nine months later, during a fellow-up interview, the mother expresses her satisfaction with the outcomes of the conference: both her sons have almost fulfilled their debts and made other friends. Another side effect is that professionals are no longer involved in this situation.

## 5. Discussion

### 5.1. The Alienated Individual and the Need for a Cultural Environment

The presented case demonstrates the difficulty of developing authority and loyalty in a composed family of kin and non-kin ties. It also shows how a life-world led intervention, such as FGC, helps to widen the social circle and opens up clogged channels of solidarity. In sticking to a modest role, the social worker displayed a remarkable view on his profession by facilitating social work performed by divergent actors and thus encouraging thick solidarity within the extended family. He appeared well-aware of the fact that state agencies can hinder solidarity and unintentionally reproduce dependency on professionals.

Long before Robert Putnam documented the atomisation of society, in Bowling Alone [6], Nisbet [51] described how the state erodes the roles of associations, social institutions and communities. Although slowly transforming, social institutions can be seen as cultural produced patterns that already existed before we were born, which were delivered to us by previous generations and which will still exist when we are gone. Examples of such institutions are language, family, marriage, friendship, law, religion, education and economy. Institutions are natural, tacit and powerful sources of regulation, which provide a stable cultural environment, alleviating people from uncertainty [52]. The characteristics of institutions have both an encouraging and a discouraging effect. Loyalties, bonds, routines and traditions produce security and continuity, but also limit freedom of choice.

The unbridled behaviour of the sons in the highlighted case can be viewed as a lack of moral upbringing, but also as an erosion of a particular social institution. The sons and their parents seemed not sufficiently embedded in a wider social system. The regulatory properties of structures beyond the person appear powerless. The individual family members might be liberated from all sorts of obligations and traditions, but they did not stand in for one another and did not make an effort to maintain the integrity of their shared life. An environment that provides physical, psychological and socio-cultural needs, remains crucial for survival. In the case of this family, a wider support network, enacted by extended family members, was required, to meet the demands of life. FGC, opened up the possibility of calling in these ‘auxiliary troops’. The case illustrates the downsides of freedom, horizontalisation and the lack of authority that comes with it. Institutional theory does not, however, explain what is really going on. The presented sociological theories and historical insights that we have shared so far are helpful, but not sufficient, for understanding the need for the strengthening of primary groups. Therefore, the sphere theory of Peter Sloterdijk [53,54,55] is brought in.

### 5.2. Co-Isolation in Bubbles and Spheres

Sloterdijk describes how people surround themselves with spatial spheres (their home, a community, a nation) to gain a sense of feeling being protected against others and against the world. People seek protection in their houses, parks and nations, but also in intimate relationships. The sphere that people share in intimate relationships forms a barrier against outside dangers and offers solidarity for those inside. All relationships based on solidarity are, in Sloterdijk’s view, sphere formations. But, in the world of expanding globalisation, the synthesis of community, religion and politics in one great monosphere is evaporated and transforms into a “super tribalistic psychological community”, where everyone increasingly ends up in a state of co-isolation [54]. People are living in ‘bubbles’ that are separated from each other by foam. Through internet and social media, spheres of immunity against dangers from the outside are built with likeminded others. In the presented case, the foam in between the bubbles of the sons and their parents, envisioned a herd of independents, desperately in search for commonalities against a background of total atomisation—even the search for commonalities itself is ambivalent. Sloterdijk describes that such bubbles are doomed to burst apart.

The sphere theory of Sloterdijk helps us to understand the difficulties of families and communities in addressing social problems. It reveals that solidarity is only felt for those who share the same sphere and that even this commitment is ambivalent. It also helps in understanding why a focused intervention, like FGC, that has no other ambition beyond strengthening the problem-solving of the primary group, holds promise for succeeding. The sphere theory makes it understandable why grown-up children and their parents, such as those presented in the case study, can easily lose each other. It reveals why the loss of solidarity between the disaffected sons and their uncertain parents impaired the immunity of the whole family—Even to such an extent that the state agencies nearly took over. The case illustrates that, in isolated bubbles, it is hard for parents to connect with their adult children as they live in their own sphere. Moreover, when parents are not embedded in a supportive community themselves, it is also hard for them to learn the art of parenting from other parents.

Even the civil society and professional society themselves are co-isolated and locked up in separate bubbles. FGC, and other life-world led interventions, can offer a platform to join the forces and regain solidarity on a small-scale. In a way, these interventions can be seen as an attempt to accomplish Habermas’ ideal speech situation. In the described case, the colonisation of the life-world by the system-world was not only pushed back, redressing the balance between the life-world and system-world, but also a dialogue and common understanding between the two worlds emerged in a concrete situation. The social worker, therefore, could stay aloof, which encouraged the primary group to make their own plan.

It is likely that the state would have intervened with legal actions when the family in the presented case did not have an established plan. That the plan succeeded in this case meant a shift of power from a state agency to the family. In other words, the colonisation of the life-world by strategic action of the system-world was prevented.

## 6. Conclusions

In the presented case, and in other cases studied, the shared life events of precarity do not always lead to thick solidarity, nor do their relationships appear as enduring entities. The recent studies into FGC disclose complex forms of sacrifice and self-interest, of solidarity and calculation, of generosity and obligatory giving and of intimacy and aloofness. Do life-world led interventions, like FGC, invoke new forms of loyalties, involvement and social embeddedness and therefore, perhaps less freedom? In the presented case, the sons became aware of their moral obligations, mutual respect was restored and the freedom to do whatever they wanted was limited. Some sort of ownership was, nevertheless, established; everyone, including the sons, had contributed to the decision-making process when they took part in the family group conference.

In the introduction of this paper, the question was raised of how the need for life-world interventions, like FGC, could be understood. As sketched, the rise of social security, civil rights and horizontal relationships brought individual freedom, but also weakened kin ties. The protective features of larger supportive familial networks, nonetheless, are crucial to survive in contemporary Western societies, while state agencies only have a limited capacity to strengthen these networks.

A social intervention, which aims to strengthen of primary groups, with a specific, but modest, ambition, such as FGC, contributes to small-scale solidarity. This ambition is not inclined to establish broad social cohesion within society, but to restore immunity (protection) and solidarity in primary groups, resolving issues with those (family, neighbours, colleagues) who share a sphere (a situation, a process, a fate). The need for life-world led interventions in contemporary Western societies is understandable, considering the weakening of kin ties and the loss of thick solidarity within and between social groups, while the rational and calculating methods of the system-world do not have the potential to strengthen the communities at stake, nor to alleviate the co-isolation of individuals and families. Its productive power stems from a shift of power from state agencies to families and communities, from public to private spheres and from the professional to the civil society. In widening social circles and opening up clogged channels of solidarity, life world-led interventions help primary groups to meet the challenges connected to the ambivalent commitment of individualistic cultures in contemporary Western societies.
